# Mucinous cholangiocarcinoma: Clinicopathological features of the rarest type of cholangiocarcinoma

**DOI:** 10.1002/ags3.12016

**Published:** 2017-06-07

**Authors:** Tatsuaki Sumiyoshi, Yasuo Shima, Takehiro Okabayashi, Ayako Ishikawa, Manabu Matsumoto, Jun Iwata, Sojiro Morita, Taijiro Sueda

**Affiliations:** ^1^ Department of Gastroenterological Surgery Kochi Health Sciences Center Kochi Japan; ^2^ Department of Gastroenterology Kochi Health Sciences Center Kochi Japan; ^3^ Department of Diagnostic Pathology Kochi Health Sciences Center Kochi Japan; ^4^ Department of Radiology Kochi Health Sciences Center Kochi Japan; ^5^ Department of Surgery Applied Life Sciences Institute of Biomedical and Health Sciences Hiroshima University Hiroshima Japan

**Keywords:** intraductal papillary neoplasm of the bile duct, intrahepatic cholangiocarcinoma, mucinous cholangiocarcinoma

## Abstract

Mucinous cholangiocarcinoma is extremely rare and its clinicopathological features remain unclear. The present study aimed to analyze published data on mucinous cholangiocarcinoma. Medical databases were searched from 1980 to 2016, and clinicopathological data for 16 mucinous cholangiocarcinoma patients were obtained. Characteristic imaging findings, including hypovascular tumor with peripheral enhancement on computed tomography and angiography, extremely high intensity on T2‐weighted magnetic resonance images, intratumoral calcification and luminal communication between the tumor and bile duct on cholangiography, were noted. Mucinous cholangiocarcinoma was correctly diagnosed in one patient only, with some patients diagnosed as low‐malignant biliary cystic tumors preoperatively. Five cases were followed up after the first medical examination, and three of these were initially diagnosed as biliary cystadenoma or intraductal papillary neoplasm of the bile duct. All five tumors showed marked enlargement within 4 months of follow up. Macroscopically, the resected tumors were non‐cystic/solid in seven cases, and cystic in seven. Tumor diameter ranged from 5 cm to 22 cm, and mucoid cut surface, lobulation, lack of capsule and papillary growth were observed. Microscopically, co‐existing intraductal papillary neoplasm of the bile duct was noted in three of five patients with available data. Nine of 10 cases in whom the pathological stage was reported had advanced disease with lymph node and/or distant metastasis, and 5‐year survival was achieved in one microinvasive case only. Overall 1‐ and 3‐year survival rates were 60.1% and 40.1%, respectively. The possibility of mucinous cholangiocarcinoma should be considered when biliary cystic tumors are detected on imaging modalities, despite the rarity of this tumor.

## 1 INTRODUCTION

Mucinous carcinoma is defined in the presence of large extracellular mucus lakes containing floating carcinoma cells, accounting for more than 50% of neoplasm.^1^ This entity has been well defined in the breast, colon, rectum, and pancreas.^1^, ^2^, ^3^ However, the clinicopathological characteristics of mucinous cholangiocarcinoma have not been elucidated owing to the rarity of this tumor. In a series of cases reported by Nakajima et al.,^4^ there was only one mucinous cholangiocarcinoma among 102 consecutive cases of intrahepatic cholangiocarcinoma. In fact, most previous reports on mucinous cholangiocarcinoma were case reports, and no detailed review on this disease exists. Thus, we conducted a retrospective systemic review of mucinous cholangiocarcinoma cases, including one patient treated in our institution, to elucidate the clinicopathological characteristics of this rare tumor.

## 2 METHODS

### 2.1 Eligibility criteria

The current systemic review was carried out according to the Preferred Reporting Items for Systematic reviews and Meta‐Analyses statement.^5^ Observational studies written in English or Japanese were eligible for inclusion. However, articles in Japanese without an English abstract were excluded. All procedures carried out in studies involving human participants were in accordance with the ethics committee of the Kochi Health Sciences Center and with the Declaration of Helsinki.

### 2.2 Literature search

A literature search was carried out using the following terms: “mucinous cholangiocarcinoma,” “mucinous carcinoma of intrahepatic bile duct,” and “colloid carcinoma in liver.” The MEDLINE and Igakuchuo‐Zashi database, a database of Japanese articles with English abstracts, were searched from 1980 to 2016. Studies in the reference lists of the retrieved articles were also searched. By reading the articles in detail, confusing biliary tumors such as biliary cyst adenoma or intraductal papillary neoplasm of the bile duct were carefully excluded. Cases in which mucus lakes were definitely described were considered eligible.

### 2.3 Data collection and assessment

The following data were extracted from the identified studies: the patient characteristics, value of tumor markers, imaging findings, pretreatment diagnosis, treatment and prognosis.

### 2.4 Statistical analysis

Survival rates were generated using the Kaplan‐Meier method. Data were analyzed by using SPSS v.19 software (IBM, Armonk, NY, USA).

## 3 RESULTS

### 3.1 Report of a case from our institution

**Figure 1:**
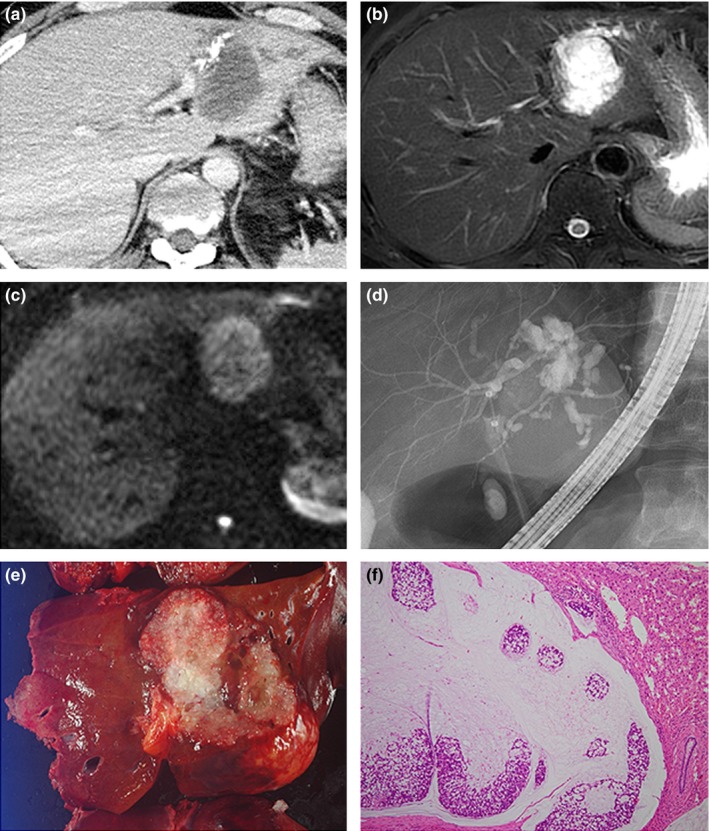
Imaging and pathological findings of the case treated in our institution. (A) Contrast‐enhanced computed tomography shows a low‐density tumor with peripheral calcification in the lateral segment of the liver. (B, C) Magnetic resonance imaging of the liver tumor shows extremely high intensity on T2‐weighted images (B), and high intensity on diffusion‐weighted images (C). (D) Endoscopic retrograde cholangiography shows communication between the intrahepatic bile duct and interior of the liver tumor. No mucus plug was observed in the bile duct. (E) The cut surface of the resected specimen shows a well‐demarcated, whitish‐to‐translucent, solid mucus mass. (F) Microscopically, numerous mucinous lakes containing floating eosinophilic neoplastic cells are observed.

A 49‐year‐old man presented with severely impaired consciousness and was admitted to our institution. Whole‐body computed tomography (CT) scanning showed subarachnoid bleeding and a 5.4‐cm‐sized low‐density tumor with peripheral calcification in the lateral segment of the liver (Figure 1A). Carcinoembryonic antigen (CEA) and carbohydrate 19‐9 (CA19‐9) levels were 2.9 ng/mL and 7.5 U/mL, respectively, which were within the normal ranges. After brain aneurysm clipping, the patient underwent several imaging tests for the liver tumor. Magnetic resonance imaging (MRI) showed low intensity on T1‐weighted (T1W) images, extremely high intensity on T2W images, and high intensity on diffusion weighted images (DWI) (Figure 1B, C). Endoscopic retrograde cholangiography showed communication between the intrahepatic bile duct and interior of the tumor (Figure 1D). The extra‐heptatic bile duct showed no dilatation, and neither mucus plug nor biliary stones were detected. Bile cytology showed no sign of malignancy. Biliary cystic tumor, especially intraductal papillary neoplasm of the bile duct (IPNB), was suspected. Although surgery was considered the most appropriate treatment option, close follow up was selected considering the need for rehabilitation for the aphasia and hemiparesis after the stroke. After 2 months of follow up, the CEA and CA19‐9 values had increased from 2.9 ng/mL and 7.5 U/mL to 3.8 ng/mL and 9.7 U/mL, respectively. Contrast‐enhanced CT showed tumor growth from 5.4 cm to 7.6 cm in diameter over 2 months. Consequently, surgery was planned, and left hepatectomy concomitant with left caudate lobe resection and regional lymph node dissection was carried out. The cut surface of the resected specimen showed a well‐demarcated, whitish‐to‐translucent, solid mucus mass (Figure 1E). Microscopically, numerous mucinous lakes containing floating eosinophilic neoplastic cells were observed (Figure 1F). IPNB was not observed in the involved bile duct. Multiple lymph node metastases were confirmed in the hepatic and suprapancreatic glands. According to the UICC‐TNM classification system, the cholangiocarcinoma was classified as Stage IVA (T1N1M0). Oral S‐1 (TS‐1; Taiho Pharmaceutical, Tokyo, Japan) chemotherapy was given post‐surgery and, at the latest follow‐up (3 months post‐surgery), the patient showed no sign of recurrence.

### 3.2 Literature review

Fourteen eligible articles were identified from the electronic databases search.^6–19^ No systemic review was included. Except for one article,^15^ all of the remaining articles were case reports. One article described biliary cystic tumors, and included two cases of mucinous cholangiocarcinoma.^15^ Finally, comprehensive data on 16 cases of mucinous cholangiocarcnoma, which included the case treated in our institution, were gathered.

### 3.3 Clinical characteristics

Clinical characteristics of the 16 patients are shown in Table 1. The patient population comprised eight males and eight females with a median age of 57.5 years (range, 33‐78 years). Hepatitis B virus antigen and hepatitis C virus antibody findings were described in five cases. Hepatitis B virus antigen was negative in all patients whereas the hepatitis C virus antibody was positive in one patient. In six patients, previous histories of biliary disease were noted, including choledocholithiasis in three patients, hepaticolithiasis in one, cholelithiasis in one, cholangitis in two, primary sclerosing cholangitis (PSC) in one, extrahepatic bile duct cancer in one, and pancreaticobiliary maljunction in one. In 11 patients, clinical symptoms were noted; these included abdominal pain in four patients, jaundice in two, fever in two, nausea in one, lumbago in one, abdominal discomfort in one, abdominal mass in one, skin nodules in one, and abdominal distention in one. In the remaining five patients, no clinical symptoms existed.

**Table 1:** Reported cases of mucinous cholangiocarcinoma

Case	Author	Year	Age (years)	Sex	HBsAg/ HCV‐Ab	Previous history	Symptoms
1	Mottoo et al.^6^	1993	65	M		Encephalomyelitis	Nausea
2	Sasaki et al.^7^	1995	49	F		Choledocholithiasis	Lumbago
3	Sonobe et al.^8^	1995	78	M	–/–	ND	Jaundice
4	Chow et al.^9^	1997	41	M		Cholangitis, choledocholithiasis	Abdominal pain, fever
5	Nishiyama et al.^10^	1997	73	F		Cholelithiasis, choledocholithiasis	None
6	Gotoh et al.^11^	1999	33	F	–/–	None	None
7	Mizukami et al.^12^	1999	74	M		ND	Jaundice
8	Bu‐Ghanim et al.^13^	2004	50	F		PSC, cholangitis, UC	Abdominal pain, fever
9	Matsuda et al.^14^	2005	69	M		Gastric ca, extrahepatic BDC	Abdominal discomfort
10	Zen et al.^15^	2006	66	M		ND	Abdominal pain
11	Zen et al.^15^	2006	52	F		ND	Abdominal pain
12	Vernez et al.^16^	2007	41	F		MTS, colon polyp, endometrial polyp	Abdominal mass, skin nodules^a^
13	Oshiro & Esaki^17^	2011	63	F		PBM, hepaticolithiasis	Abdominal distension
14	Kang et al.^18^	2012	75	F	–/–	HT	None
15	Kai et al.^19^	2013	51	M	–/+	DM, chronic hepatitis C	None
16	Our case	2016	49	M	–/–	SAH	None

^a^Two skin nodules located in the bilateral retro‐auricular areas.

BDC, bile duct cancer; ca, cancer; DM, diabetes mellitus; F, female; HBsAg/HCVAb, hepatitis B surface antigen/hepatitis C antibody; HT, hypertension; M, male; MTS, Muir‐Torre syndrome; ND, not described; PBM, pancreaticobiliary maljunction; PSC, primary sclerosing cholangitis; SAH, subarachnoid hemorrhage; UC, ulcerative colitis.

### 3.4 Tumor markers

CEA and CA19‐9 levels were most commonly measured, and elevated values of CEA and CA19‐9 were noted in six of 11 (54.5%) and in six of nine patients (66.7%), respectively (Table S1). Values of CA19‐9 >500 U/mL were noted in five of nine patients (55.5%). Increases in the CEA and CA19‐9 values were observed within 4 months of follow‐up in three and one patients, respectively. Elevated values of sialyl SSEA‐1 antigen, cancer antigen 72‐4, squamous cell carcinoma antigen and neuron‐specific enolase were also noted, although these values were measured only in one or two patients each. Elevated values of alpha‐fetoprotein and protein induced by vitamin K absence/antagonist‐2 were noted in one patient. However, this patient was diagnosed with mucinous cholangiocarcinoma showing features of hepatocellular carcinoma (case 15). Values of cancer antigen 125, detection of a pancreatic cancer‐associated antigen‐2, and NCC‐ST‐439 were within the normal range.

### 3.5 Imaging findings

Imaging findings of the mucinous cholangiocarcinomas are summarized in Table 2. The tumors appeared hyperechoic and hypoechoic in two patients each. Calcification was observed in one patient. CT showed a low‐density tumor in eight patients, lobulation in three, calcification in three, peripheral enhancement in three, and portal vein thrombus in two patients. Intrahepatic bile duct dilatation only, without a mass, was observed in one patient. On MRI, the tumor showed low intensity on T1W images in seven patients, high intensity on T2W images in eight, and high intensity on diffusion‐weighted images (DWI) in one patient. Cholangiography showed luminal communication between the tumor and bile duct in four patients, extrahepatic bile duct dilatation in two, mucin secretion in one, and dilated papilla in one patient.

**Table 2:** Imaging findings of the included cases

Imaging modality (n)	Image findings (n)
US (4)	Hyperechoic lesion (2), hypoechoic lesion (2), calcification (1)
CECT (11)	Low‐density tumor (8), lobulation (3), calcification (3), peripheral enhancement (3), portal vein thrombus (2), no mass (1)
MRI (8)	T1W, low intensity (7); T2W, high intensity (8); DWI^a^, high intensity (1)
Angiography (4)	Hypovascular tumor (2), hypervascular at the margin (2)
Cholangiography^b^ (6)	Communication between the tumor and bile duct (4), extrahepatic bile duct dilatation (2), dilated papilla (1), mucin secretion (1)

^a^This imaging finding was described in one case only from our institution.

^b^Endoscopic retrograde cholangiography or percutaneous transhepatic cholangiography.

CECT, contrast‐enhanced computed tomography; DWI, diffusion weighted image; MRI, magnetic resonance imaging; T1W, T1‐weighted image; T2W, T2‐weighted image; US, ultrasonography.

### 3.6 Diagnosis and treatment

Mucinous cholangiocarcinomas were located in the right lobe in nine patients, in the left lobe in six, and in both lobes in one patient (Table 3). Maximum tumor diameter on imaging studies ranged from 6.4 cm to 14.6 cm (median, 8.4 cm). Rapid tumor growth within 2 months was observed in two patients (cases 1, 2). Maximum tumor diameter of the resected specimen ranged from 5 cm to 22 cm (median, 7.5 cm).

**Table 3:** Diagnoses and treatment of the included cases

Case	Location	Size^a^ (interval)	Size^b^	Pretreatment diagnosis	Treatment
1	Rt	4→9 (4 wks)	ND	ND	Hepatectomy
2	Rt (ant)	3.5→6.4 (2 mo)	9.5	Mucinous cholangiocarcinoma	Cisplatin chemotherapy
3	Rt (ant)	ND	5.0	(Autopsy case)	None
4	Rt	ND	6.0	(Autopsy case)	None
5	Lt (lat)	12.4	ND	ICC	Lt hepatectomy & PD
6	Lt	7.5	8.0	ICC	Lt hepatectomy, LN dissection
7	Both	ND	ND	Adenocarcinoma of the bile duct	None
8	Rt (post)	ND	5.5	PSC	Liver transplantation → PD^c^
9	Lt (lat)	7.0	7.0	Metastatic liver tumor	Lateral segmentectomy → RT
10	Lt (med)	ND	ND	ND	Lt hepatectomy
11	Lt (med)	ND	ND	ND	Lt hepatectomy & choledochectomy
12	Rt	ND	22.0	ND	Rt hepatectomy
13	Rt	12→14 (4 mo)	ND	BCAC	Rt hepatectomy & adrenalectomy
14	Rt	12→14.6 (2 mo)	13.0	BCA or BCAC	Rt hepatectomy
15	Rt	ND	13.4	HCC	Rt hepatectomy → S‐1
16	Lt (lat)	6→7.8 (3 mo)	5.7	IPNB	Lt hepatectomy, LN dissection → S‐1

^a^Tumor size (cm) on imaging studies. ^b^Tumor size (cm) in the resected specimen. ^c^PD 3 days after the transplantation.

ant, anterior segment; BCA/BCAC, biliary cystadenoma/cystadenocarcinoma; HCC, hepatocellular carcinoma; ICC, intrahepatic cholangiocarcinoma; IPNB, intraductal papillary neoplasm of the bile duct; lat, lateral segment; LN, lymph node; Lt, left; med, medial segment; mo, months; ND, not described; PD, pancreaticoduodenectomy; post, posterior segment; PSC, primary sclerosing cholangitis; RT, adjuvant radiation therapy; Rt, right; S‐1, S‐1 adjuvant chemotherapy; wks, weeks.

Pretreatment diagnosis was intrahepatic cholangiocarcinoma in two patients, biliary cystadenoma or adenocarcinoma (BCA or BCAC) in two, metastatic liver tumor in one, IPNB in one, adenocarcinoma of the bile duct in one, and hepatocellular carcinoma in one. Two cases of mucinous cholangiocarcinoma (cases 3, 4) were detected on autopsy, and one case (case 8) was detected in the resected specimen after liver transplantation for PSC. Mucinous cholangiocarcinoma was correctly diagnosed before treatment by needle biopsy in one patient only.

Regarding treatment, hepatectomy was carried out in 12 patients, including one liver transplantation for a PSC patient. Adjuvant radiation therapy to the anterior mediastinum was carried out in one patient (case 9), and adjuvant S‐1 chemotherapy was carried out in two patients (cases 15, 16). No treatment for mucinous cholangiocarcinoma was done in three patients, including two autopsy cases. Cisplatin chemotherapy was carried out in one patient.

Macroscopic findings of the resected tumor varied in each case. The cut surface of the tumor was non‐cystic/solid in seven cases, and cystic in seven cases. Type of cyst was unilocular in two cases and multilocular in five. Mucoid cut surface, lobulation, lack of capsule and papillary growth were noted in 10, three, three, and three patients, respectively. Microscopically, the presence or absence of IPNB, which is reported to be the precancerous lesion of mucinous cholangiocarcinoma, was noted in five patients, and the lesion was observed in three patients. Presence or absence of ovarian‐like stroma was examined in four patients, and this lesion was not observed in any patient.

### 3.7 Prognosis

Lymph node metastases were noted in five of six patients (Table 4). Distant metastases were noted in five of 10 patients. The site of distant metastasis was the lymph nodes in two patients, lungs in two, peritoneum in one, skin in one, and adrenal gland in one patient. Nine of 10 cases in whom the pathological stage was disclosed were classified as Stage IV, whereas the remaining case was classified as Stage III according to the UICC grading system. Nine of these 10 cases were found to have advanced disease with lymph node metastasis and/or distant metastasis. Tumor recurrence was noted in four patients, and the timing of recurrence was 5, 8, 10 and 11 months after surgery. Recurrence sites included the humerus, thoracic vertebrae, brain, and liver. Treatment after recurrence was radiation therapy for bone metastasis in one patient and tumorectomy of a metastatic brain tumor in one patient. Survival data were available for 14 patients. Of these, five patients died as a result of mucinous cholangiocarcinoma within 1 year. Overall 1‐ and 3‐year survival rates were 60.1% and 40.1%, respectively. Five‐year survival was achieved in one microinvasive case only.

**Table 4:** Prognoses of the included cases

Case	UICC stage	Recurrence	Timing of recurrence (no. months after surgery)	Recurrent site	Treatment after recurrence	Survival (months)	Outcome
1	TxNxM1 (PER) Stage IVB	+	ND	ND	None	4	DD
2	TxN1M1 (PUL, LYM) Stage IVB	(No surgery^a^)				5	DD
3	TxN1M0 Stage IVA	(Autopsy case)				3	DD
4	TxNxM1 (PUL, LYM) Stage IVB	(Autopsy case)				ND	ND
5	ND	+	11	Bone^b^	RT	15	AD
6	T1N1M0 Stage IVA	–				12	RFS
7	ND	(No surgery^a^)				0	DD
8	TxN1M0 Stage IVA	ND				ND	ND
9	T3N0M0 Stage III	+	5	Bone^c^	None	7	DD
10	ND (microinvasive)	–				134	RFS
11	ND (microinvasive)	–				28	RFS
12	TxNxM1 (SKI) Stage IVB	+	10	Brain	Tumorectomy	10	AD
13	TxNxM1 (ADR) Stage IVB	–				6	RFS
14	ND	–				6	RFS
15	ND	+	8	Liver	None	26	DD
16	T1N1M0 Stage IVA	–				3	RFS

^a^No surgery as a result of advanced disease or poor general condition. ^b^Humerus. ^c^Thoracic vertebrae.

AD, alive with recurrent disease; ADR, adrenals; DD, death by primary disease; LYM, lymph nodes; ND, not described; PER, peritoneum; PUL, pulmonary; RFS, relapse‐free survival; RT, radiation therapy; SKI, skin.

## 4 DISCUSSION

Mucinous cholangiocarcinoma is the rarest type of cholangiocellular carcinoma,^12^, ^20^ and this carcinoma is reported to be derived from IPNB.^15^, ^16^, ^21^ IPNB is pathologically characterized by prominent intraductal papillary proliferation with delicate fibrovascular cores and frequent mucin hypersecretion,^15^, ^16^, ^21^ and it is regarded as the biliary counterpart of intraductal papillary mucinous neoplasm of the pancreas. When accompanied by invasive lesions, IPNB is known to be associated with conventional tubular adenocarcinoma or mucinous cholangiocarcinoma.^22^, ^23^, ^24^ IPNB is characterized by the intestinal phenotype (mucin2 [MUC2]+/cytokeratin20+), with the carcinogeneses leading to tubular adenocarcinoma and mucinous cholangiocarcinoma associated with increasing MUC1 expression and MUC1‐negativity, respectively.^21^

In the current study, 13 of 16 mucinous cholangiocarcinomas were reported from Japan and Korea, suggesting that Asian patients might have a predisposition to develop this tumor. A previous history of biliary disease and chronic hepatitis C was noted in seven patients. Chronic inflammation of the biliary epithelium has been reported to be a risk factor for IPNB^22^, ^23^, ^25^, ^26^ and conventional cholangiocarcinoma,^27^ and this finding might thus also be applicable to mucinous cholangiocarcinoma. Chou and Chan^28^ reported that all three cases of mucinous cholangiocarcinomas in their study were heavily infested with *Clonorchis sinensis*. Zen et al. also reported that nine cases of mucinous cholangiocarcinoma were detected in 110 cases of surgically resected hepatolithiasis,^21^ although these cases were not included in the present study because of insufficient clinical information.

In terms of tumor markers, CA19‐9 and CEA were commonly used for the diagnosis of mucinous cholangiocarcinoma, similar to conventional cholangiocarcinoma. In a series of cholangiocarcinoma patients without PSC, the sensitivity of CA 19‐9 concentrations >100 U/mL in diagnosing cholangiocarcinoma was 53%.^29^ However, although CEA is also measured frequently, it has been reported to have unsatisfactory sensitivity and specificity for cholangiocarcinoma.^30^, ^31^ In the current study, elevated values of CEA were noted in 54.5% of mucinous cholangiocarcinoma patients. CEA, as well as CA19‐9, might hence be a useful marker for mucinous cholangiocarcinoma.

On diagnostic imaging, ultrasonography showed varying findings in each case. Mucinous cholangiocarcinoma was hyperechoic in two cases, but was conversely hypoechoic in another two cases. Mottoo et al. and Hayashi et al. reported that mucinous carcinoma appeared similar to cavernous hemangioma on ultrasonography.^6^, ^20^ The diversity of ultrasonography findings might be because of differences in the gross finding of this tumor. In the current study, solid and cystic tumors comprised half of the cases each, and the cystic tumors could further be divided into unilocular and multilocular types. This variation of gross finding might be as a result of the amount of mucin in the tumor. In solid, non‐cystic mucinous cholangiocarcinoma, the cut surfaces were reported to be “mucinous, mucoid, translucent, and gelatinous”, indicating mucin production. If the proportion of mucin in the tumor was large, it might appear to be a cystic tumor. In contrast, if the proportion was small, the tumor might appear to be a solid tumor.

In contrast, the CT and MRI findings were relatively consistent. The mucinous cholangiocarcinomas tended to be shown as hypodense tumors with peripheral enhancement on contrast‐enhanced CT, and this finding coincided with the angiographic appearance. On T2W MRI images, the tumor tended to be shown as an extremely high‐intensity lesion, owing to the influence of the abundant mucin in the tumor.^20^ This tumor has been reported to have no fibrous capsule and to directly invade the adjacent hepatic parenchyma^8^ and, in this study, it was found to occasionally invade the portal vein, resulting in portal vein tumor thrombus (cases 7, 15). Characteristic imaging findings of mucinous cholangiocarcinoma ware as follows: (i) a hypovascular tumor with peripheral enhancement on CT and angiography; (ii) extremely high intensity on T2 W MRI images; (iii) intratumoral calcification; and (iv) luminal communication between the tumor and the bile duct on cholangiography. One peculiar finding is that mucus secretion was confirmed only in one of six cholangiography images, despite of the presence of mucus lake and luminal communication between the bile duct. The frequency of mucus secretion into the bile duct was low, and it might be one of the causes of difficult early detection of this tumor.

The diagnosis of mucinous cholangiocarcinoma is difficult because of the rarity of this tumor. The biggest problem is that the tumor can be easily diagnosed as a low‐malignant biliary cystic tumor. In the current study, five cases were followed up after the first medical examination, and three of these five cases were initially diagnosed as biliary cystadenoma or IPNB. All five tumors showed visual enlargement within 4 months of follow up, and the tumor diameter grew from 4 cm to 9 cm in just 4 weeks in the most rapidly growing case (case 1). When diagnosing biliary cystic tumors, careful exclusion of mucinous cholangiocarcinoma using the characteristic imaging findings might be necessary, despite the rarity of this tumor.

The prognosis of mucinous cholangiocarcinoma has been estimated to be poor because of the difficulty of early detection of this tumor. As mentioned earlier, nine of 10 patients in whom the pathological stage was disclosed were found to have advanced disease with lymph node and/or distant metastasis, and 5‐year survival was achieved only in one microinvasive case. Sasaki et al.^7^ described the characteristics of mucinous cholangiocarcinoma as rapid growth, widespread metastases, and poor prognosis, in comparison to the characteristics of IPNB. Conversely, Zen et al.^21^ reported mucinous cholangiocarcinoma as a less advanced tumor than conventional cholangiocarcinoma. One hundred and ten surgically resected specimens for hepatolithiasis were pathologically examined in Zen's series, and nine cases of mucinous cholangiocarcinoma were detected out of 19 cholangiocaricnomas with IPNB. The tumor size ranged from 1.2 cm to 3.5 cm, and no cases showed lymph node or distant metastases. However, hepatectomy was carried out for hepatolithiasis in their series and, as a result, mucinous cholangiocarcinomas were detected in the resected specimens. Therefore, these nine mucinous cholangiocarcinoma cases are considered not to show the real clinical characteristics of this tumor, although the correlation between hepatolithiasis and mucinous cholangiocarcinoma is interesting.

The present study had a serious limitation. The number of cases was small as a result of the rarity of this tumor. However, the current study describes the clinicopathological features of mucinous cholangiocarcinoma in detail, including image findings, diagnosis, treatment and prognosis.

In conclusion, the current study investigated the clinicopathological features of the extremely rare entity, mucinous cholangiocarcinoma. The possibility of mucinous cholangiocarcinoma should be considered when biliary cystic tumors are detected on imaging modalities.

## DISCLOSURE

Conflict of Interest: Authors declare no conflict of interests for this article.
