# Supplementary material for: Mucinous cholangiocarcinoma: Clinicopathological features of the rarest type of cholangiocarcinoma
Source: Ann Gastroenterol Surg. 2017 Jun 7;1(2):114–21. doi: 10.1002/ags3.12016 (PMC5881371; doi:10.1002/ags3.12016)
Supplement: Supplementary file 1 [file AGS3-1-114-s001.docx]

Supplemental Table 1 Tumor marker levels in the included cases

| Case | CEA  (<5.0 ng/ml) | CA19-9  (<37U/ml) | SLX  (<38 U/ml) | CA72-4  (<4.0 U/ml) | SCC  (<1.5 ng/ml) | NSE  (<10.0 U/ml) | AFP  (<20 ng/ml) | PIVKA2  (<4 mAU/ml ) | CA125  (<25 U/ml) | DUPAN-2  (<150 U/ml) | NCC-ST-439  (<7.0 U/ml) |
| --- | --- | --- | --- | --- | --- | --- | --- | --- | --- | --- | --- |
| 1 | **125→1000^a^** | 35 | **47** | **71** |  |  |  |  | 20 | 72 | 6.8 |
| 2 | **33.3** | **820** |  |  |  |  | Normal |  |  |  |  |
| 3 | 1.4 |  |  |  |  |  | 1.0 |  |  |  |  |
| 5 | **20.4** | **1388** |  |  |  |  |  |  |  |  |  |
| 6 | <1 | 16 | **49** |  | **2** | **11.7** |  |  | 18 |  |  |
| 8 |  | **595** |  |  |  |  |  |  |  |  |  |
| 9 | 4.9 | **1393.5** |  |  |  |  |  |  |  |  |  |
| 12 | **983** |  |  |  |  |  |  |  |  |  |  |
| 13 | **16.9→25.8^b^** |  |  |  |  |  |  |  |  |  |  |
| 14 | 2.44 | **22.39** |  |  |  |  | 3.25 |  |  |  |  |
| 15 | **6.4** | **540** |  |  |  |  | 76.3 | 5750 |  |  |  |
| 16 | 2.9→3.8^c^ | 7.5→9.7 |  |  |  |  | 2.0 | 22 |  |  |  |

All tumor marker values were measured before the treatment. **Bold characteristics** indicate abnormally elevated levels of markers. CEA: carcinoembryonic antigen, CA19-9: carbohydrate 19-9, SLX: sialyl SSEA-1 antigen, CA72-4: cancer antigen 72-4, SCC: squamous cell carcinoma antigen, NSE: neuron-specific enolase, AFP: alpha-fetoprotein, PIVKA2: protein induced by vitamin K absence/antagonist-2, CA125: cancer antigen 125, DUPAN-2: detection of a pancreatic cancer-associated antigen-2.

^a^1 month interval, ^b^4 months interval, ^c^2 months interval.
